# Clinical reasoning in managing chronic hip pain: One in two Australian and New Zealand physiotherapists diagnosed a case vignette with clinical criteria for hip OA as hip OA. A cross‐sectional survey

**DOI:** 10.1002/msc.1751

**Published:** 2023-03-02

**Authors:** Travis Haber, Rana S. Hinman, Fiona Dobson, Bill Vicenzino, Ben Darlow, Sam Kayll, Michelle Hall

**Affiliations:** ^1^ Centre for Health, Exercise and Sports Medicine Department of Physiotherapy School of Health Sciences The University of Melbourne Victoria Australia; ^2^ School of Health and Rehabilitation Sciences University of Queensland Saint Lucia Australia; ^3^ Department of Primary Health Care and General Practice University of Otago Wellington Wellington New Zealand

**Keywords:** chronic pain, evidence based practice, hip conditions

## Abstract

**Objectives:**

Using a case vignette of an adult (George) presenting with hip pain consistent with hip OA, this study aimed to describe: (a) whether physiotherapists make diagnoses and identify bodily structures using either patient history and/or physical examination findings; (b) which diagnoses and bodily structures physiotherapists attribute to the hip pain; (c) how confident physiotherapists were in their clinical reasoning using patient history and physical examination findings; (d) what treatments physiotherapists would offer to George.

**Methods:**

We conducted a cross‐sectional online survey of physiotherapists in Australia and New Zealand. We used descriptive statistics to analyse closed questions and content analysis for open‐text responses.

**Results:**

Two hundred and twenty physiotherapists completed the survey (39% response‐rate). After receiving the patient history, 64% diagnosed George's pain and 49% of these as hip OA; 95% attributed George's pain to a bodily structure(s). After receiving the physical examination, 81% diagnosed George's hip pain and 52% of these as hip OA; 96% attributed George's hip pain to a bodily structure(s). Ninety‐six percent of respondents were at least somewhat confident in their diagnosis after the patient history, and 95% were similarly confident after the physical examination. Most respondents offered advice (98%) and exercise (99%), but fewer offered treatments for weight loss (31%), medication (11%), and psychosocial factors (<15%).

**Discussion:**

About half of the physiotherapists that diagnosed George's hip pain made a diagnosis of hip OA, despite the case vignette including clinical criteria for a diagnosis of OA. Physiotherapists offered exercise and education, but many physiotherapists did not offer other clinically indicated and recommended treatments, such as weight loss and sleep advice.

## 1 INTRODUCTION

Chronic hip pain affects up to one in three people aged 50 or over (Christmas et al., 2002; Murphy et al., 2010; Segal et al., 2007; Sundén‐Lundius et al., 2005), resulting in personal disability and high societal costs (Australian Institute of Health and Wellness, 2019; Haber et al., 2022). Hip osteoarthritis (OA) among middle‐aged and older adults is a common condition causing hip pain (Jordan et al., 2009; Murphy et al., 2010). Hip OA is the leading cause of total hip replacements, and rates of both are rising – driving unsustainable increases in healthcare costs (Cross et al., 2014; Pabinger et al., 2018). Optimizing the nonsurgical management of hip OA is thus essential. Physiotherapists are key health professionals assessing, diagnosing, and providing nonsurgical treatments for people with hip OA (Victorian Musculoskeletal Clinical Leadership Group, 2018).

The National Institute for Health and Care Excellence (NICE) recommends that health professionals diagnose a person with hip OA if they are 45 years or older and have activity‐related joint pain – medical imaging is not required to confirm this diagnosis (National Institute of Health and Care Excellence, 2022). However, diagnosing hip pain is complex. Health professionals must often differentiate systemic conditions (e.g. rheumatoid arthritis) and other hip and back conditions that co‐exist or share overlapping symptoms (e.g. glutaeal tendinopathy) (Aresti et al., 2016; Calders & Van Ginckel, 2018; Grimaldi et al., 2015; Metcalfe et al., 2019; Reiman et al., 2020; Reiman et al., 2014; Woodley et al., 2008). Furthermore, as individual clinical tests often cannot rule in or rule out a condition confidently (Griffin et al., 2016; Metcalfe et al., 2019; Reiman et al., 2014, 2020), health professionals likely need to consider a range of assessment findings when diagnosing hip pain.

Physiotherapists should determine the probability of a diagnosis of hip pain using findings from a patient history (e.g. age, self‐reported pain using stairs) and adjust that probability using further information from a physical examination (e.g. reduced hip internal rotation) (Metcalfe et al., 2019). Failing to adequately consider all available clinical information can lead to diagnostic errors, potentially driving suboptimal care (Balogh et al., 2015; Cook & Décary, 2020). While physiotherapists should tailor treatments to a range of factors (e.g. patient preferences) (Lin et al., 2020), evidence and guidelines recommend treatments specific to a diagnosis – and recommended treatments can differ between diagnoses of hip pain (Bannuru et al., 2019; Griffin et al., 2016; Grimaldi et al., 2015; Kemp, Mosler et al., 2020; Kemp, Risberg et al., 2020; Kolasinski et al., 2020; National Institute of Health and Care Excellence, 2022). It is thus important to know if physiotherapists diagnose a person with hip OA from a case vignette, which comprehensively details the patient history and physical examination findings and includes clinical criteria for a diagnosis of OA. We identified no surveys ascertaining which diagnoses physiotherapists would attribute to a patient with undiagnosed hip pain (Barber et al., 2022; Maxwell et al., 2021; Slade et al., 2016).

While clinical guidelines recommend that health professionals determine a diagnosis for hip pain (Lin et al., 2020; National Institute of Health and Care Excellence, 2022), it is unclear if physiotherapists believe it is essential to diagnose hip pain in order to manage it. Indeed, health professionals differ in their views of diagnosing joint pain in the shoulder and back (Cools & Michener, 2017; Kent & Keating, 2004; Lewis & Powell, 2022). For example, physiotherapists may diagnose shoulder pain as a syndrome (shoulder impingement syndrome), an observed impairment (e.g. flexibility deficit), or a less‐specific label (rotator cuff related shoulder pain), or attribute it to a structural pathology (i.e. rotator cuff tear) (Cools & Michener, 2017; Lewis & Powell, 2022; Zadro et al., 2021). Clinical reasoning about diagnosing hip pain is thus further complicated by the fact that it could similarly be condition‐specific (e.g. hip OA), nonspecific (e.g. hip pain), or a structural pathology (e.g. bursitis) (Enseki et al., 2014; Metcalfe et al., 2019).

Attributing hip pain to a structural pathology may influence physiotherapists' treatments. For example, pathoanatomic approaches to care (e.g. attributing joint pain to damaged and worn‐out cartilage) may drive fear of movement among people with joint pain and can lead to an over‐emphasis on a patient's physical rather than psychosocial needs (e.g. failing to address emotional distress) (Bunzli et al., 2017; Christe et al., 2021; Egerton et al., 2017; Gardner et al., 2017; Haber et al., 2022; de Oliveira et al., 2020; Teo et al., 2020; Bunzli et al., 2019). While physiotherapists often deliver guideline‐recommended treatments for hip pain, such as education and exercise (Cowan et al., 2010; French, Woodley, et al., 2019; Holden et al., 2018; Stephens et al., 2019), less is known about whether they offer treatments addressing other psychosocial needs of people with hip pain (Haber et al., 2022), such as sleep and mental health issues (Barten, Dorsman, Dekker, Veenhof, & de Bakker, 2015; Cowan et al., 2010; French, Grimaldi, et al., 2019; Holden et al., 2018; Stephens et al., 2019).

Using a case vignette of an adult (George) presenting with hip pain consistent with hip OA, this study aimed to describe: (a) whether physiotherapists make diagnoses and identify bodily structures using either patient history and/or physical examination findings; (b) which diagnoses and bodily structures physiotherapists attribute to the hip pain; (c) how confident physiotherapists were in their clinical reasoning using patient history and physical examination findings; (d) what treatments physiotherapists would offer to George.

## 2 MATERIALS AND METHODS

### 2.1 Study design

We conducted a cross‐sectional online survey across Australia and New Zealand. We reported this study according to the Strengthening the Reporting of Observational Studies in Epidemiology (STROBE) guideline (Von Elm et al., 2014).

### 2.2 Sample

We recruited physiotherapists who manage chronic hip pain in Australia and New Zealand between November 2020 and March 2022. We recruited through advertisements with the Australian Physiotherapy Association, Physiotherapy New Zealand, special interest groups (i.e. Physiotherapy New Zealand Sports and Exercise Physiotherapy and New Zealand Manipulative Physiotherapists Association), social media platforms (i.e. Twitter, Instagram, Facebook, and LinkedIn), and direct contact with relevant bodies and businesses. To encourage participation, all participants could opt‐in to enter the draw to win an Apple iPad Air. Participants were eligible to participate if they were registered as a physiotherapist in Australia or New Zealand, and if, in the last 6 months, they had managed at least one adult, 45 years or older, with a primary complaint of chronic hip pain.

### 2.3 Survey development

By collaborating with physiotherapists researching chronic hip pain, we developed an online survey with a hypothetical case vignette (see supplementary digital content 1 for the online Survey) in Qualtrics (Provo, UT, USA). The survey asked physiotherapists to answer questions about George, a 55‐year‐old adult with chronic hip pain and co‐existing low back pain (see supplementary digital content 2 for the case vignette). Surveys using case vignettes accurately capture clinical practices when measured against standardized patients and clinicians' self‐reported clinical practices (Peabody et al., 2004; Rutten et al., 2006). Consistent with past studies using case vignettes (Holden et al., 2018), we developed our case vignette based on actual patients seen by an author (TH) in a private physiotherapy practice in Melbourne, Australia. Seven physiotherapists with experience clinically managing or researching chronic hip pain reviewed the case vignette and piloted the survey. Based on their feedback, we clarified minor details in the case vignette and altered the format of one question. In addition, three middle‐aged or older adults with lived experiences of chronic hip pain and its management reviewed the case vignette. These consumers confirmed the vignette accurately captured their experiences of chronic hip pain. The case vignette had a Grade 7 readability (see https://readabilityformulas.com/free‐readability‐formula‐tests.php), which is optimal for communication content in health (Sharma et al., 2020).

The survey consisted of three groups of questions: (a) the *most probable* contributing bodily structures to George's hip pain; (b) the *most probable* clinical syndrome, diagnosis or health condition describing George's hip pain; (c) management and treatment of George. The survey included closed questions (with multiple‐choice and Likert scale response options) and open‐text responses. We designed the survey to first provide respondents information from the patient's (George's) history, allowing them to form hypotheses in their clinical reasoning (i.e. bodily structures and diagnoses), and then provide further information with findings from George's physical examination to see if physiotherapists used this information to modify their hypotheses or confidence in them.

### 2.4 Data collection and analyses

We presented the case vignette and collected the data via Qualtrics (Provo, UT, USA) and used Microsoft Excel (Microsoft Corporation, Washington, US) for analysis. Descriptive statistics were used to describe the participants' characteristics, clinical history, and management of the case vignette and chronic hip pain. Data pertaining to statements of the survey were described as numbers (percentages).

One author (TH) performed a content analysis (Hsieh & Shannon, 2005; Stemler, 2000) of data from open‐entry text boxes. The author (TH) inductively categorized open‐text responses based on diagnoses, conditions, syndromes, and structural pathologies of the hip. The author (TH) then refined these categories by grouping existing categories that captured similar data. For example, the diagnoses ‘hip labral tear’, ‘hip degeneration’, and ‘’hip cartilage tears’ were combined under a new category ‘hip joint structural pathology’. Furthermore, while hip OA encompasses structural pathology at the hip, we did not consider specific structural pathologies (e.g. ‘hip labral tear’) as a diagnosis of hip OA, as OA is now best understood as a whole joint disease (Hunter & Bierma‐Zeinstra, 2019). We determined that the trustworthiness of the content analysis was greater than 90% for open‐text responses – we determined this by calculating the concordance of two authors (TH and SK) in applying the coding framework to 20% of the open text.

## 3 RESULTS

### 3.1 Survey responses and demographic and clinical characteristics

Screening questions were completed by 714 people, with 571 (79%) determined eligible. Of those eligible to participate, we obtained data for demographic and clinical characteristics for 471 (80%) respondents, with 220 (39%) respondents completing the survey. Table 1 summarises participant demographic and clinical characteristics. Just over half of the respondents were male (55%), and most worked in private practice (83%) in a metropolitan area (73%) in Australia (76%). Most participants (77%) managed chronic hip pain in middle‐aged or older adults up to 5 times per week.

**TABLE 1:** Demographic and clinical characteristics of respondents, completing (*n* = 220) and not completing the study (*n* = 251), reported as mean (%) unless otherwise indicated.

Characteristics		Complete survey responses n (%)	Incomplete survey responses n (%)
Gender	Female	98 (45)	169 (68)
	Male	120 (55)	80 (32)
	Non‐binary	1 (<1)	1 (<1)
	Prefer not to say	1 (<1)	1 (<1)
Age (yrs)		37 (11.8)^b^	37 ± (11.5)^b^
Place of physiotherapy education	Australia	176 (81)	189 (75)
	New Zealand	25 (11)	20 (8)
	India	4 (2)	11 (4)
	United Kingdom of Great Britain and Northern Ireland	5 (2)	15 (6)
	Finland	1 (<1)	5 (2)
	Hong Kong	1 (<1)	3 (1)
	Ireland	1 (<1)	2 (1)
	Netherlands	1 (<1)	3 (1)
	South Africa	1 (<1)	5 (2)
	Philippines	0 (0)	2 (1)
	Angola	0 (0)	1 (<1)
	Chile	0 (0)	1 (<1)
	Hong Kong	0 (0)	1 (<1)
	Ireland	0 (0)	1 (<1)
	Uruguay	0 (0)	1 (<1)
Years working as physiotherapists managing chronic hip pain	0–5 years	53 (24)	72 (29)
	6–10 years	41 (19)	40 (16)
	11–15 years	37 (17)	42 (17)
	16–20 years	24 (11)	26 (10)
	21–25 years	16 (7)	24 (10)
	26–30 years	19 (9)	22 (9)
	More than 30 years	30 (14)	24 (10)
Work setting	Private practice	182 (82)	180 (72)
	Hospital	23 (10)	46 (18)
	Community centre	8 (4)	9 (4)
	Residential aged care facility	7 (3)	16 (6)
Geographical remoteness	Major city	161 (73)	Not available
	Rural	59 (27)	
Country of work	Australia	191 (76)	Not available
	New Zealand	29 (24)	11 (4)
Clinical hours worked per weeks	1–10 h	11 (5)	31 (12)
	11–20 h	31 (14)	56 (25)
	21–30 h	49 (22)	112 (44)
	31–40 h	109 (50)	29 (12)
	>40 h	20 (9)	
Patients managed per week with chronic hip pain 45 years or older	0‐5 patients/week	170 (77)	198 (79)
	6‐10 patients/week	42 (19)	45 (18)
	11‐20 patients/week	7 (3)	7 (3)
	>20 patients/week	1 (<1)	1 (<1)
Physiotherapists with postgraduate qualifications^a^	Clinical doctorate	7 (3)	9 (4)
	No postgraduate qualification relating to physiotherapy	120 (55)	127 (51)
	MSc musculoskeletal therapy	27 (12)	27 (11)
	MSc sports physiotherapy	30 (14)	20 (8)
	MSc manipulative therapy	10 (5)	6 (2)
	MSc other	6 (3)	7 (3)
	PhD	6 (3)	9 (4)
	Postgraduate diploma	35 (16)	61 (24)
	Other postgraduate diploma	16 (7)	31 (12)
Physiotherapists completing postgraduate training in management of chronic hip pain	Yes	60 (27)	69 (27)
	No	160 (73)	182 (73)

^a^ Values may equal greater than participant numbers as participants could select multiple options.

^b^ Standard deviation.

There were differences between those completing the survey and those who did not (Table 1). More females (68%, *n* = 169) than males (32%, *n* = 80) dropped out. In addition, about double the proportion of respondents working in an aged facility did not complete the survey (*n* = 16, 6%) compared with completing it (*n* = 3, 7%).

### 3.2 The *most probable* clinical syndrome, diagnosis, or health condition

#### 3.2.1 After receiving George's patient history (see supplementary digital content 2 for the case vignette)

**FIGURE 1:**
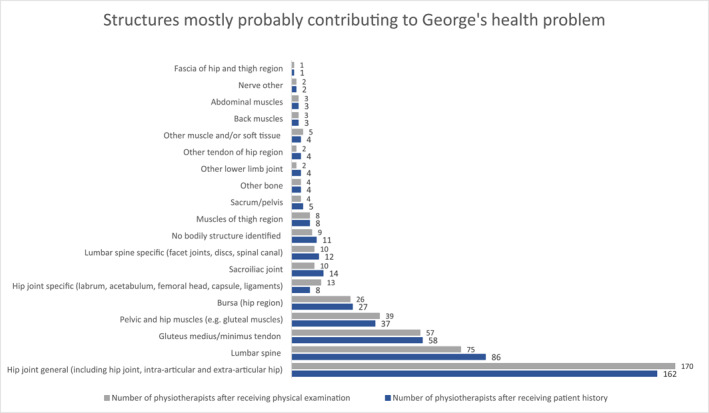
Most probable clinical syndrome, diagnosis or health condition for George's hip pain identified by physiotherapists in open text format, including physiotherapists that provided no diagnosis. Numbers represent the number of times the category was identified by a physiotherapist (*n* = 220).

After receiving George's patient history, 36% of 220 respondents (*n* = 79) indicated it was unimportant or very unimportant to diagnose George's health problem at this point of the consultation; these respondents did not provide a diagnosis. Fifty‐four percent (*n* = 119) of respondents indicated it was important or very important, and 10% (*n* = 22) were uncertain whether, to diagnose George's health problem as a specific clinical syndrome, diagnosis, or health condition. Using open‐text to provide only one response, these respondents (*n* = 141, 64%) most commonly diagnosed George's health problem as hip OA (*n* = 69, 49%), glutaeal tendinopathy (*n* = 22, 16%), or hip joint structural pathology (including labral tear, cartilage tear, degenerative changes) (*n* = 17, 13%) (Figure 1). Almost all respondents diagnosing George's health problem (*n* = 134, 96%) were at least somewhat confident in their diagnosis (i.e. diagnosis, syndrome, or condition).

#### 3.2.2 After receiving George's physical examination

After receiving George's physical examination, 19% (*n* = 42) of 220 respondents indicated it was unimportant or very unimportant to diagnose George's health problem (see supplementary digital content 3 for full ratings of importance); these respondents did not provide a diagnosis for it. Sixty‐seven percent of respondents (*n* = 148) indicated it was important or very important, and 14% were uncertain (*n* = 30) whether, to diagnose George's health problem as a specific clinical syndrome, diagnosis, or health condition. Similarly to after the patient history, the most common diagnoses made by respondents (*n* = 174, 81%) were: hip OA (*n* = 93, 52%), hip joint structural pathology (including labral tear, cartilage tear, degenerative changes) (*n* = 24, 13%), and glutaeal tendinopathy (*n* = 23, 13%) (Figure 1). Most respondents (*n* = 169, 95%) diagnosing George's health problem were at least somewhat confident in their diagnosis (i.e. diagnosis, syndrome, or condition) (see supplementary digital content 4 for full confidence ratings).

Of those respondents (*n* = 141) that diagnosed George's health problem after receiving the patient history (Part A): 86% (*n* = 121) did not change their diagnosis of George's hip pain after receiving the physical examination, and 93% (*n* = 112) of whom did not change their level of confidence in their diagnosis of George after receiving the physical examination.

### 3.3 The *most probable* contributing bodily structures

#### 3.3.1 After receiving George's patient history (see supplementary digital content 2 for the case vignette)

**FIGURE 2:**
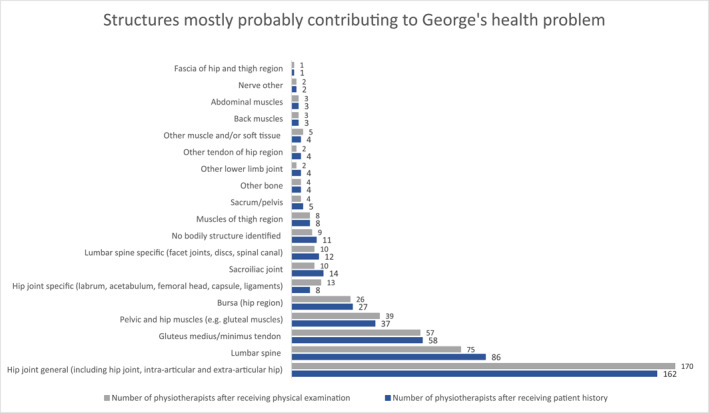
Categories of bodily structures that physiotherapists identified as most probably contributing to George's hip pain using open text format, including those not identifying a bodily structure. Numbers represent the number of times the category was identified by a physiotherapist (*n* = 211). Physiotherapists could identify more than one bodily structure.

After receiving George's patient history, 5% of 220 respondents (*n* = 12) indicated it was unimportant or very unimportant to determine which bodily structure(s) were contributing to George's health problem at this point of the consultation; these respondents did not attribute a bodily structure to George's health problem. Ninety‐five percent of respondents (*n* = 208) indicated it was important or very important to determine which bodily structure(s) contributed to George's health problem. Using open text format, these respondents (*n* = 208, 95%) most commonly identified the hip joint (*n* = 162, 78%), lumbar spine (*n* = 86, 42%), glutaeal medius/minimus tendon (*n* = 57, 27%) as the most probable bodily structures contributing to George's health problem (Figure 2). Respondents could indicate more than one structure. Almost all respondents (*n* = 200, 98%) who identified a bodily structure(s) were at least somewhat confident that they determined the most probable bodily structure contributing to Georges' health problem.

#### 3.3.2 After receiving George's physical examination

After receiving both George's patient history and physical examination information, 4% (*n* = 9) of 220 respondents indicated it was unimportant or very unimportant to determine which bodily structure(s) were contributing to George's health problem; these respondents did not attribute a bodily structure to George's health problem. Ninety‐six percent of respondents (*n* = 211) indicated it was important or very important to determine which bodily structure(s) were most probably contributing to George's health problem. Similarly to after the patient history, the most commonly identified structures by respondents (*n* = 211, 96%) were: hip joint (*n* = 170, 81%), lumbar spine (*n* = 75, 36%), glutaeal medius/minimus tendon (*n* = 58, 24%) (Figure 2). Almost all respondents (*n* = 204, 98%) were at least somewhat confident that they identified the most probable bodily structure contributing to Georges' health problem.

Of the respondents (*n* = 208) identifying a bodily structure after receiving the patient history (Part A): 85% (*n* = 177) did not change their opinion after receiving George's physical examination, and 92% of whom (*n* = 163) did not change their confidence in the structure(s) that they identified after receiving George's physical examination.

### 3.4 Management and treatment of George

**FIGURE 3:**
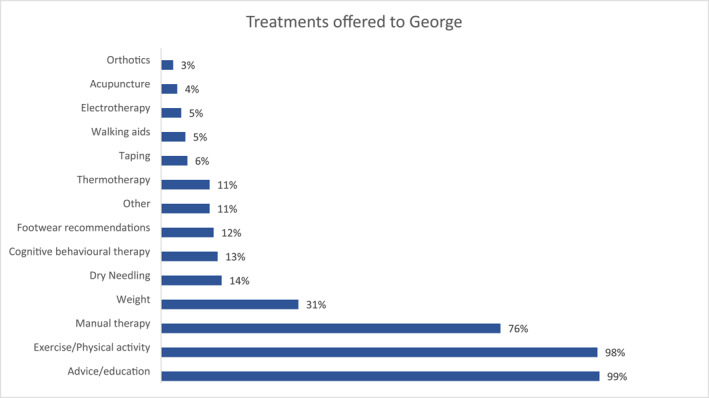
Treatments offered to George in the first two consultations by physiotherapists – expressed as a percentage (*n* = 220).

Respondents were asked to select (from a list) up to 3 treatments they would offer within the first two consultations. The 3 treatments that respondents most often offered to George were: (a) advice and education (*n* = 217, 99%); (b) exercise and/or physical activity prescription (*n* = 216, 98%); and 3) manual therapy (*n* = 168, 76%) (Figure 3). The top 3 things participants educated George about were: (a) knowledge about the condition (*n* = 169, 78%); (b) load management (*n* = 155, 71%); and (c) physiotherapy treatment options (*n* = 70, 32%). The top 3 forms of exercise and/or physical activity prescription participants prescribed were: (a) strength training (*n* = 150, 69%); (b) general physical activity (*n* = 92, 43%); and (c) joint mobility (*n* = 81, 38%) (see supplementary digital content 5 for full details of advice/education, exercise, weight loss, and manual therapy offered).

## 4 DISCUSSION

Based on the self‐reported management of a case vignette (George), this study highlights that most physiotherapists believe it is important to diagnose a patient with hip pain. Despite the case vignette including patient history and physical examination findings consistent with NICE clinical criteria for OA, only about half of the physiotherapists that made a diagnosis converged on a diagnosis of hip OA (National Institute of Health and Care Excellence, 2022). Most physiotherapists attributed bodily structures to George's chronic hip pain, including the whole joint (hip and back) and specific structures such as tendons and bursa. Finally, most physiotherapists offered George exercise, education, and manual therapy. Fewer respondents offered treatments specifically targeting psychosocial factors, weight loss, and analgesics – despite being clinically indicated treatments based on the case vignette.

Although George's assessment information included clinical criteria for a diagnosis of hip OA, only 52% of physiotherapists made this diagnosis (53% including the diagnosis of combined hip and back OA). There was also substantial variability across diagnoses (see Figure 1); we identified 12 categories of diagnoses made by physiotherapists, with many incorporating multiple diagnoses (e.g. hip joint structural pathology). The complexities of differentiating diagnoses at the hip might have contributed to some of this variability —many conditions at the hip share overlapping symptoms or co‐exist, which can span multiple body locations (Aresti et al., 2016; Grimaldi et al., 2017; Metcalfe et al., 2019; Reiman et al., 2020). For example, glutaeal tendinopathy (the third most common diagnosis in this study) and hip OA can both present with lateral hip pain (Fearon et al., 2014; Grimaldi et al., 2015; Lesher et al., 2008). Physiotherapists may thus need to consider the baseline probability for a range of diagnoses (i.e. based on the prevalence of a diagnosis among similar patient groups) and then adjust the likelihood of these diagnoses based on a range of assessment findings (Balogh et al., 2015; Cook & Décary, 2020). It is important to acknowledge that we sought to capture these clinical challenges in our case vignette.

Almost half of the respondents made diagnoses that either lack evidence‐based diagnostic criteria (e.g. structural pathologies at the back (Hartvigsen et al., 2018) or require additional information, such as specific tests or investigations (e.g. single leg stance test for glutaeal tendinopathy (Grimaldi et al., 2017), which were not provided as part of the vignette. Based on the best available evidence (Katz et al., 2021; Metcalfe et al., 2019), information from the patient history and physical examination in our case vignette would increase the likelihood that George had hip OA. Diagnostic errors are common across healthcare and partly due to the following reasons: difficulties with interpreting test results; biases leading clinicians to form strong beliefs (i.e. anchoring bias) about a diagnosis based on early information (e.g. from a patient history); and biases leading clinicians to favour information that confirms a certain diagnosis (i.e. confirmation bias) (Berner & Graber, 2008; Cassam, 2017; Cook & Décary, 2020; Meyer et al., 2013; Scott & Crock, 2020; Singh et al., 2017; Verghese et al., 2015).

Overconfidence can also underlie diagnostic errors (Berner & Graber, 2008; Cassam, 2017). We found that almost all respondents were at least somewhat confident in their diagnosis after receiving the patient history, and many did not change their confidence after receiving physical examination findings. Health professionals often underestimate rates of diagnostic errors, overestimate certainty provided by assessment findings, and are more confident than accurate in making a diagnosis (Berner & Graber, 2008; Cassam, 2017; Meyer et al., 2013; Scott & Crock, 2020). To enhance care for people with hip OA, physiotherapists may need strategies and cognitive training to enhance their diagnostic skills, such as diagnostic checklists with debiasing strategies and training in analytical thinking (Ely et al., 2011; Myung et al., 2013; Scott & Crock, 2020; Shimizu et al., 2013). However, further research may also be needed to address gaps and limitations in the evidence for diagnosing hip pain. It is unclear which combination of information and test results could best identify those with a condition (e.g. likelihood ratios for hip OA or glutaeal tendinopathy) compared to those without it (Griffin et al., 2016; Grimaldi et al., 2017; Metcalfe et al., 2019; Reiman et al., 2020). There are also limitations with generalizing the existing evidence base (i.e. clinical test metrics such as likelihood ratios) to patients with hip pain within community or primary health care (Metcalfe et al., 2019).

Physiotherapists attributed George's hip pain to bodily structures ranging from the whole joint (i.e. hip and back) to specific structural pathologies (e.g. tendons and bursas). Similarly, most physiotherapists made a whole‐joint diagnosis (e.g. hip OA), and about 1 in 3 diagnosed it as a specific structural pathology (e.g. bursitis). Diagnosing or identifying specific structural pathologies is of uncertain relevance for managing hip OA. Although people with joint pain desire a precise diagnosis (Barber et al., 2022; Maxwell et al., 2021; Slade et al., 2016), bony joint changes and soft tissue changes (e.g. tendon or bursa) at the hip pain are present in people with and without hip pain (Kim et al., 2015; Woodley et al., 2008). Hip OA is now best understood as a disease of the whole joint (Hunter & Bierma‐Zeinstra, 2019). Thus, while identifying the structural cause of hip pain may inform tailored treatment for some hip conditions, incorrectly identifying structural pathology could lead to unnecessary or non‐recommended treatments for OA (e.g. advice for unloading specific to glutaeal tendon pathology (Grimaldi et al., 2015) but not OA (National Institute of Health and Care Excellence, 2022)).

Despite about 1 in 2 physiotherapists not diagnosing George with hip OA, we found that physiotherapists were broadly delivering recommended treatments for it (Bannuru et al., 2019; Kolasinski et al., 2020; National Institute of Health and Care Excellence, 2022; Victorian Musculoskeletal Clinical Leadership Group, 2018); these clinical practices are also largely consistent with those for hip OA in the UK and Australia (Cowan et al., 2010; Holden et al., 2018). Almost all respondents offered George exercise, most often strength training, and education, commonly about the condition and load management, combined with manual therapy. However, as aspects of education are condition‐specific (French et al., 2015; National Institute of Health and Care Excellence, 2022), some physiotherapists may have better tailored their education to George by making a diagnosis of OA. Conversely, weight loss and medication advice, conditionally recommended treatments for OA (Bannuru et al., 2019; Kolasinski et al., 2020; National Institute of Health and Care Excellence, 2022) – George was overweight and had only used a non‐recommended analgesic (paracetamol) – were offered by less than 1 in 10 physiotherapists. Respondents also infrequently offered treatments specifically targeting psychosocial factors. For example, physiotherapists advised about load management 10 times more than about sleep management – sleep advice is recommended for people with OA when indicated (Geenen et al., 2018; Victorian Musculoskeletal Clinical Leadership Group, 2018), and George had sleep issues articulated in the patient history. It is possible that more physiotherapists would have offered these treatments to George if they had diagnosed him with hip OA; however, further research is required as our cross‐sectional survey was not designed to test this hypothesis.

There are several possible explanations for these treatment practices. Non‐steroidal anti‐inflammatory drugs reduce pain similarly to exercise for people with OA (Katz et al., 2021). Thus, physiotherapists may have prioritized exercise over medication advice. Furthermore, more research is needed to determine if psychosocial treatments and weight loss benefit people with hip OA (Bannuru et al., 2019; Ho et al., 2019). Lastly, physiotherapists have previously reported knowledge or skills gaps and low confidence in delivering psychosocial, medication and weight loss interventions; some also believe these treatments may fall outside their scope of practice (Allison et al., 2019; Briggs et al., 2019; Cowell et al., 2018; Driver et al., 2021; Gardner et al., 2017; Holden et al., 2018; Holden, Waterfield, et al., 2019; Holden, Whittle, et al., 2019; Nielsen et al., 2014; Synnott et al., 2015).

### 4.1 Strengths and limitations

This survey used a robust methodology, including open and closed questions to collect mixed‐methods data. We aimed to capture clinical practices more accurately by using a case vignette that did not specify a diagnosis and by including open questions (Peabody et al., 2004; Rutten et al., 2006). This case vignette was adapted from real patients and was reviewed by consumers with lived experiences of hip pain and physiotherapists with experience managing hip OA. However, possibly due to the time commitment involved (20–40 min) – needed to explore the iterative clinical reasoning for diagnosing hip pain – only about one in three eligible people completed the survey. We do not know why more females than males dropped out of the survey; we explored if differences in work setting explained these findings but found no evidence it did. Physiotherapists working in aged care were also less likely to finish the survey, and thus, findings from this study may not generalise to this setting. Another limitation was our sample size, representing a small percentage of possible physiotherapists across Australia and New Zealand. It is unknown how many physiotherapists in these countries manage chronic hip pain. Lastly, there may be limitations with generalizing this study's results: clinical practices for a hypothetical situation may not match actual clinical practices, and the results of this study may only apply to physiotherapists working in Australia and New Zealand.

## 5 CONCLUSION

About half of the physiotherapists that diagnosed George's hip pain made a diagnosis of hip OA, despite the case vignette including clinical criteria for a diagnosis of OA. Most respondents were confident in their diagnosis using the patient history only, and their confidence remained unchanged with information about a physical examination. While most physiotherapists offered exercise and education, it is possible that physiotherapists could better tailor education if they identified a diagnosis of OA. Furthermore, despite being clinically indicated, many physiotherapists did not offer other conditionally recommended treatments for George, such as treatments focussing on weight loss and sleep.

## AUTHOR CONTRIBUTION

Travis Haber: Study conception and design; data collection; data analysis; manuscript consultation and writing. Rana Hinman: Study conception and design; manuscript consultation and writing. Fiona Dobson: Study conception and design; manuscript consultation and writing. Bill Vicenzino: Study design; manuscript consultation and writing. Ben Darlow: Study design; manuscript consultation and writing. Sam Kayll: data analysis; manuscript writing. Michelle Hall: Study conception and design; data analysis; manuscript consultation and writing.

## CONFLICT OF INTEREST

None declared.

## ETHICS STATEMENT

The Melbourne University Institutional Human Ethics Committee approved the study (ethics ID: 2057929.1), and participants provided informed consent.

## Supporting information

Supplementary Material

Supplementary Material

Supplementary Material

Supplementary Material

Supplementary Material

## Data Availability

Data available on request.
