# Supplementary material for: Clinical reasoning in managing chronic hip pain: One in two Australian and New Zealand physiotherapists diagnosed a case vignette with clinical criteria for hip OA as hip OA. A cross‐sectional survey
Source: Musculoskeletal Care. 2023 Mar 2;21(3):763–75. doi: 10.1002/msc.1751 (PMC10947065; doi:10.1002/msc.1751)
Supplement: Supplementary file 1 — Supplementary Material [file MSC-21-763-s001.pdf]

Chronic Hip Pain Survey

Start of Block: Block 10

Q1 We are interested in how physiotherapists manage patients with chronic hip pain aged 45 years and over. By chronic hip pain we mean hip and/or groin pain and associated symptoms that have been present for more than 3 months. Management refers to all aspects of your clinical care - assessment of a patient, implementation of a management plan (including treatment and advice), and coordination of continuity of care - within your professional scope of practice as a physiotherapist.

Q2 Are your currently working as a registered physiotherapist in either Australia or New Zealand?

- ○ Yes (1)
- ○ No (2)

Skip To: End of Survey If Are your currently working as a registered physiotherapist in either Australia or New Zealand? = No

---

Q3 In your role as a physiotherapist, in the last 6 months have you managed **at least** one patient with a primary complaint of chronic hip pain who was aged 45 years or over?

- ○ Yes (1)
- ○ No (2)

Skip To: End of Survey If In your role as a physiotherapist, in the last 6 months have you managed at least one patient wit... = No

---

Q4
Based on your answers you are eligible to participate in this study. Please read the (click here) Plain Language Summary If you would like more details regarding this survey you

can email Travis Haber at t.haber@student.unimelb.edu.au.
Do you understand the information contained within the Plain Language Summary?

- ◯ Yes (30)
- ◯ No (31)

Skip To: End of Survey If Based on your answers you are eligible to participate in this study. Please read the (click her... = No

End of Block: Block 10

Start of Block: Block 1: Participant Characteristics

Q5 Please state your **age** (**in numbers only)**:

____

Q6 Please choose your gender:

- ◯ Male (1)
- ◯ Female (2)
- ◯ Non-Binary (3)
- ◯ Prefer not to say (4)

Q7 Please select the country where you received your physiotherapy qualification:

▼ Australia (9) ... Zimbabwe (195)

Q8 Please select the number of **years** that you have been **working** as a physiotherapist that has included the management of patients with chronic hip pain:

- ○ 0-5 years (4)
- ○ 6-10 years (5)
- ○ 11-15 years (6)
- ○ 16-20 years (7)
- ○ 21-25 years (8)
- ○ 26-30 years (9)
- ○ More than 30 years (10)

Q9 Please select your current primary site of clinical practice:

- ○ Private Practice (1)
- ○ Community Health Center (2)
- ○ Residential Aged Care Facility (3)
- ○ Hospital (4)

---

*

Q10 Please state the postcode of your primary place of clinical practice (**in numbers only**):

____

Q11 Please select the numbers of clinical hours that you currently work per a week:

- ○ 1-10 hours (2)
- ○ 11-20 hours (3)
- ○ 21-30 hours (4)
- ○ 31-40 hours (5)
- ○ >40 hours (6)

Q12 Do you have any postgraduate qualifications relating to physiotherapy?

- ☐ I do not have a postgraduate qualification relating to physiotherapy (18)
- ☐ Postgraduate diploma (10)
- ☐ MSc sports physiotherapy (11)
- ☐ MSc musculoskeletal therapy (12)
- ☐ MSc manipulative therapy (13)
- ☐ MSc other (14)
- ☐ Clinical doctorate (15)
- ☐ PhD (16)
- ☐ Other postgraduate diploma (17)

____

Q13 Have you completed post graduate training relating to the assessment, management, or coordination of continuity of care for chronic hip pain in the past 2 years?

- Yes (1)
- No (2)

Q14 Please select how many patients you manage a week with chronic hip pain that are 45 years or older:

- 0-5 patients/week (1)
- 6-10 patients/week (2)
- 11-20 patients/week (3)
- >20 patients/week (4)

End of Block: Block 1: Participant Characteristics

Start of Block: Clincal Case - Patient History

Q15 **Survey Overview** We are asking you to consider a clinical scenario of a patient George. The survey consists of 4 parts: **Part 1:** answer a series of questions that relate to George's patient history **Part 2:** answer a series of questions that relate to George's physical examination **Part 3:** offer George treatment approaches **Part 4:** tell us your views about managing chronic hip pain At the end of the survey you will have the opportunity to **enter the draw** to **win an Apple iPad** and to ask for a **summary** of the **results**. Please consider your usual work-place setting when answering these questions. There are no right or wrong answers, we are interested in how **YOU** would manage George.

Q16 **Part 1: Patient History**

Select here to view the Patient history in a separate window [Part 1 Patient History]

Primary presenting problem:
George, a 57-year-old man, presents to you at your current site of physiotherapy clinical practice. George reports that his main problem is pain at the outside of the left hip that extends into the groin and buttock and that this pain began 3 months ago (see pain diagram). Today George rates the intensity of his hip and groin pain as a 4 out of 10 (higher scores indicate worse pain).

George's hip and groin pain started around the time he began trying to get fit for an upcoming skiing holiday (in two months' time) by walking more. His route included some stair climbing to work his thighs. He first noticed this pain on the outside of his hip when going down steps and then going up as well, and eventually when walking on the flat. The pain then spread to include the whole hip area and groin.

George is finding his sleep quality is reduced. George wakes up through the night due to his hip and groin pain. Now he is feeling more tired and irritable. After getting out of bed in the morning George reports that his hip feels stiff for approximately 30 minutes.

George has not seen any other health professional with regard to this issue. George is unsure what is causing his pain or how it happened, but believes he must have damaged something as the pain hasn't gotten any better. He is concerned that his pain won't be better by his holiday.

Secondary presenting problem:
George has a five-year history of intermittent low back pain for which he has not previously sought care. Episodes usually last for one to two months and then resolve on their own. George's back pain returned about the same time that the pain spread into his whole hip area; his back pain has gradually worsened over the last three months rather than resolving as normal. George is worried about this as the combination of back and hip pain is really wearing him down. George rates his low back pain today as 3 out of 10.

Figure: George's body chart - red shading indicates areas of pain distributions

Aggravating factors: George's hip and groin pain increase immediately when ascending or descending stairs on the left leg. George has therefore been avoiding stairs. George cannot walk more than 15 minutes continuously. Pain gets progressively worse after 5 minutes of walking and can increase to a 6 out of 10. This causes George to stop walking (to sit down). Pain starts at the outside of the left hip then spreads into the groin and bottom. George's back pain increases to a 4 out of 10 when walking. The low back pain does

not change location.   George’s hip and groin pain become more painful immediately when he puts his left shoe on. He has been avoiding this activity by wearing sandals or having his wife assist where necessary.

Easing factors: Sitting: once seated for 5 to 10 minutes after walking George’s hip and groin pain starts to lessen; it may take up to 1 hour for George's hip and groin pain to return to resting levels.   George has tried taking Panadol tablets when the pain is worse but has found this ineffective at relieving his pain.

Medical history:

George reports that: he has not had any episodes of pain referral into the legs or any changes in his lower limb sensation or strength   he has not experienced any changes with his urination and he has had no sensory disturbances around his perineal (saddle) or genital area   he has no history of cancer   he has not had any recent illness or fever   he does not smoke or drink alcohol regularly   he has not suffered from any mental illness he does not take any regular medications and has not ever had to take any regular medications   he has not had any previous lower limb injuries

George believes he is slightly overweight. He reports that he is 170cm tall and weighs 85kg.

Physical activity and exercise:

George was walking 4km every day before the hip and groin pain started. Now he is only walking incidentally around the house and at work. George enjoyed exercising at a gym once a week where he lifted weights. He ceased going to the gym after the groin and hip pain started as he thought that lifting weights may have been making the pain worse.

Social history:

George is currently working full time in an office. He has not missed any work days and he is still working normal duties (mostly desk work). He is not concerned about his work at the moment. George lives with his wife and three children in their teens. George reports that his family is supportive, but he is worried he will let them down if he can’t go on their ski holiday.

Goals and expectations:

1. George is hoping you can help reduce his pain.
2. George would like to be able to ski with his family on their upcoming holiday.

Regarding treatment, George is unsure what to expect but is happy to try what you recommend.

Q17 Select here Part 1 Patient History to open the patient history in a separate window.

End of Block: Clincal Case - Patient History

Start of Block: Block 2 Patient History Phase 1 Questions

Q18 At this point of the consultation, **how important** is it for you as the physiotherapist to determine which **bodily structure(s)** may be contributing to George's health problem?

◯ Very important (1)

◯ Important (2)

◯ Uncertain (3)

◯ Unimportant (4)

◯ Very Unimportant (5)

Display This Question:

If At this point of the consultation, how important is it for you as the physiotherapist to determin... = Very important

Or At this point of the consultation, how important is it for you as the physiotherapist to determin... = Important

Or At this point of the consultation, how important is it for you as the physiotherapist to determin... = Uncertain

Q19 Based on the patient history, what **bodily structure(s)** are you considering as the **most probable contributor(s)** to George's health problem at this point?

____

Page Break

Display This Question:

If At this point of the consultation, how important is it for you as the physiotherapist to determin... = Very important

Or At this point of the consultation, how important is it for you as the physiotherapist to determin... = Important

Or At this point of the consultation, how important is it for you as the physiotherapist to determin... = Uncertain

Q20 Please indicate how confident you are at this stage of the patient assessment that the "${Q19/ChoiceTextEntryValue}" is/are the most probable **bodily structure(s)** contributing to George's health problem:

- Not at all confident (1)
- Not very confident (2)
- Somewhat confident (3)
- Confident (4)
- Very confident (5)

Q21 At this point of the consultation, **how important** is it for you as the physiotherapist to classify George's health problem as a specific **clinical syndrome, diagnosis, or health condition**?

- Very Important (1)
- Important (2)
- Uncertain (3)
- Unimportant (4)
- Very unimportant (5)

Display This Question:

If At this point of the consultation, how important is it for you as the physiotherapist to classify... = Very Important

Or At this point of the consultation, how important is it for you as the physiotherapist to classify... = Important

Or At this point of the consultation, how important is it for you as the physiotherapist to classify... = Uncertain

Q22 Based on George's patient history, what **clinical syndrome, diagnosis, or health condition** are you considering as the **most probable** at this point for George (you can write one only)?

____

Page Break

Display This Question:

If At this point of the consultation, how important is it for you as the physiotherapist to classify... = Very Important

Or At this point of the consultation, how important is it for you as the physiotherapist to classify... = Important

Or At this point of the consultation, how important is it for you as the physiotherapist to classify... = Uncertain

Q23 Please indicate how confident you are at this stage of the patient assessment that "**${Q22/ChoiceTextEntryValue}**" is the most probable clinical syndrome, diagnosis, or health condition for George:

- ◯ Not at all confident (1)
- ◯ Not very confident (2)
- ◯ Somewhat confident (3)
- ◯ Confident (4)
- ◯ Very confident (5)

Page Break

Q29 **Part 2: The Physical Examination** Select here to view the physical examination in a separate window Part 2 physical examination

Presented below are the results of your physical examination with George. The series of questions that follow relate to this physical examination**.** Please consider your usual work-place setting.

Functional Assessment

You complete a functional assessment in the following order. You allow George to sit in between each functional assessment.

Standing posture:

In weight-bearing position no resting fixed flexion deformity observed. No resting hip rotation deformity observed.

Taking shoes on and off:

You ask George to take on and off his (laced) shoes whilst seated on a chair of standard height. When attempting to lift his foot toward himself - moving into hip flexion and external rotation - George reports stiffness and a sudden increase of pain spreading through the left hip and into the groin and bottom. He rates this pain a 6 out of 10. To complete the movement, George lowers his left foot to the ground and bends toward his foot through his spine. George's back feels stiff whilst bending to get his shoe on; he rates his low back pain a 4 out of 10 during this movement.

Gait assessment:

You observe George walking indoors on a flat surface. George reports pain during the stance phase of the left leg: at the outside of the left hip, extending through the hip and into the groin and bottom. There is an obvious limp. George reports that his hip pain increases to a 4 out of 10 when he starts walking and his back feels stiff. After walking 100 metres his hip pain is a 5 out of 10 and his back pain is a 4 out of 10. You measure George's gait speed as 1 metre per second, walking at a comfortable self-selected pace, over a 20 metre segment at the start of the gait assessment. George reports that he could have kept walking but felt his hip and groin pain were likely to get worse. This is consistent with what he normally experiences when walking.

Stairs**:**

You ask George to ascend and descend 5 standard stairs (as he normally would). George initially ascends 2 steps by leading with the right leg and stepping to with the left leg. He also uses the handrail. You ask George to alternate leading with his left and right leg without using the handrail. George can position the left leg to step up and down, but upon weight-bearing he experiences worsening pain. The pain is at the outside of the left hip, extending through the hip, and into the groin and bottom. George’s pain increases as the assessment continues. After ascending then descending the 5 stairs George rates his pain a 6 out of 10. George reports that his back felt painful when stepping with either leg; the intensity of his low back pain increased to a 5 out of 10 by the end of the assessment.

Pain after functional assessments:
George has been seated and resting for approximately 3 minutes since completing the functional assessments. Compared to before the functional assessments George reports that his hip and groin are now mildly more painful. The pain location hasn't changed but the intensity has increased to a 5 out of 10. George reports that his low back pain has increased to an intensity of 4 out of 10 and now feels stiffer whilst seated.

Lumbar assessment
Lumbar Flexion: standing 50 degrees active range of motion. George's fingertips - with straight arms - reached to his tibial tuberosity, 43cm from the ground. Stiffness and pain are the limiting factors. Pain is reported when moving through lumbar flexion at the left and right lumbar paraspinal region. No hip or groin pain reproduced. **Lumbar extension:** standing 20 degrees active range of motion. Stiffness is the limiting factor. Pain is reported on the end of range at the limit of movement. No hip or groin pain reproduced. **Lateral flexion** Left: 20 degrees active range of motion. Stiffness is the limiting factor. Pain is reported at the left paraspinal region and tightness is reported at the right paraspinal region; pain and stiffness reported on the end of range at the limit of movement. Right: 20 degrees active range of motion. Stiffness is the limiting factor. Pain is reported at the right paraspinal region and tightness is reported at the left paraspinal region; pain and stiffness reported on the end range at the limit of movement. **Lumbar quadrant test:** extension, same side lateral flexion and rotation Left: pain on end of range at the limit of movement in the left paraspinal region. No hip or groin pain reproduced. Right: pain on end of range at the limit of movement in the right paraspinal region. No hip or groin pain reproduced. **Straight leg raise test:** Left leg: 65 degrees active range of motion. George reports stiffness as the limiting factor in the hamstring and top of the calf. No hip or groin pain reproduced. Right leg: 70 degrees active range of motion. George reports stiffness as the limiting factor in the hamstring and top of the calf. No hip or groin pain reproduced. **Hip range of motion assessment:**
Hip internal rotation range of motion: supine, in 90 degrees hip and knee flexion Left hip: 10 degrees active range of motion; passive range of motion no improvement. Both pain and stiffness are the limiting factors. Pain is reported at the left groin and buttock on the end of range at the limit of movement. Right hip: active and passive range of motion within normal ranges. No pain on movement. **Hip external rotation range of motion**: supine in 90 degrees hip and knee flexion Left hip: 35 degrees active range of motion; passive range of motion no improvement. Both pain and stiffness are the limiting factors. Pain is reported at the left groin and buttock on the end of range at the limit of movement. Right hip active and passive range of motion within normal ranges. No pain on movement. **Hip abduction**: supine Left hip: active and passive range of motion within normal ranges. Pain is reported at the left groin and buttock on the end of range at the limit of movement. Right hip: active and passive range of motion within normal ranges. No pain on movement. **Hip flexion:** supine Left hip: 110 degrees active range of motion; passive range of motion no improvement. Both pain and stiffness are the limiting factors. Pain is reported at the left groin and buttock on the end of range at the limit of movement. Right hip: active and passive range of motion within normal ranges. No pain on movement. **Hip extension:** prone Left hip: active and passive range of motion within normal ranges. No pain on movement. Right hip:

active and passive range of motion within normal ranges. No pain on movement. **Tenderness on palpation**:
Painful to palpate left greater trochanter. Right side non-tender.

Q30
Select here Part 2 Physical Examination to open the physical examination in a separate window.

End of Block: Physical Examination

Start of Block: Physical examination questions

Display This Question:

If At this point of the consultation, how important is it for you as the physiotherapist to determin... = Very important

Or At this point of the consultation, how important is it for you as the physiotherapist to determin... = Important

Or At this point of the consultation, how important is it for you as the physiotherapist to determin... = Uncertain

Q31 Now that you have information from the patient history and physical examination, are you still **considering** the "**${Q19/ChoiceTextEntryValue}**" to be the **most probable bodily structure(s)** contributing to George's health problem?

○ Yes (1)

○ No (2)

Display This Question:

If Now that you have information from the patient history and physical examination, are you still co... = Yes

Q32 Are you **still** "[${Q20/ChoiceGroup/SelectedChoices}]" that the "[${Q19/ChoiceTextEntryValue}]" is/are the most probable **bodily structure(s)** contributing to George's health problem?

- ◯ Yes (1)
- ◯ No (2)

Display This Question:

If Are you still "${q://QID154/ChoiceGroup/SelectedChoices}" that the "... = No

Q33 Please indicate **how confident** you are that the "[${Q19/ChoiceTextEntryValue}]" is/are the **most probable bodily structure(s)** contributing to George's health problem:

- ◯ Not at all confident (1)
- ◯ Not very confident (2)
- ◯ Somewhat confident (3)
- ◯ Confident (4)
- ◯ Very confident (5)

Display This Question:

If At this point of the consultation, how important is it for you as the physiotherapist to classify... = Very Important

Or At this point of the consultation, how important is it for you as the physiotherapist to classify... = Important

Or At this point of the consultation, how important is it for you as the physiotherapist to classify... = Uncertain

Q34 Now that you have information from the patient history and physical examination, are you **still considering** "[${Q22/ChoiceTextEntryValue}]" as the **most probable clinical syndrome, diagnosis, or health condition** for George?

- ◯ Yes (1)
- ◯ No (2)

Display This Question:

If Now that you have information from the patient history and physical examination, are you still co... = Yes

Q35 Are you **still** "**${Q20/ChoiceGroup/SelectedChoices}**" in your **classification** of "**${Q22/ChoiceTextEntryValue}**" for George?

- Yes (26)
- No (27)

Display This Question:

If Are you still "${q://QID154/ChoiceGroup/SelectedChoices}" in your classification of "... = No

Q36 Please indicate **how confident** you are about your **classification** of "**${Q22/ChoiceTextEntryValue}**" for George?

- Not at all confident (1)
- Not very confident (2)
- Somewhat confident (3)
- Confident (4)
- Very confident (5)

Display This Question:

If Now that you have the information from the patient history and physical examination, how importan... = Very important

Or Now that you have the information from the patient history and physical examination, how importan... = Important

Or Now that you have the information from the patient history and physical examination, how importan... = Uncertain

Or Now that you have information from the patient history and physical examination, are you still co... = No

Q40 Based on George's patient history and physical examination, what **bodily structure(s)** are you considering as the **most probable contributor(s)** to George's health problem?

____

Page Break

Display This Question:

If Now that you have the information from the patient history and physical examination, how importan... = Very important

Or Now that you have the information from the patient history and physical examination, how importan... = Important

Or Now that you have the information from the patient history and physical examination, how importan... = Uncertain

Or Now that you have information from the patient history and physical examination, are you still co... = No

Q41 Please indicate how confident you are that the "**${Q40/ChoiceTextEntryValue}**" is/are the most probable **bodily structure(s)** contributing to George's health problem:

- Not at all confident (1)
- Not very confident (2)
- Somewhat confident (3)
- Confident (4)
- Very confident (5)

Display This Question:

If At this point of the consultation, how important is it for you as the physiotherapist to classify... = Unimportant

Or At this point of the consultation, how important is it for you as the physiotherapist to classify... = Very unimportant

Q42
Now that you have the information from the patient history and physical examination, **how**

important is it for you as the physiotherapist to **categorise** George's health problem as a specific **clinical syndrome, diagnosis, or health condition**?

- ○ Very important (1)
- ○ Important (2)
- ○ Uncertain (3)
- ○ Unimportant (4)
- ○ Very unimportant (5)

Display This Question:

If Now that you have the information from the patient history and physical examination, how importan... = Very important

Or Now that you have the information from the patient history and physical examination, how importan... = Important

Or Now that you have the information from the patient history and physical examination, how importan... = Uncertain

Or Now that you have information from the patient history and physical examination, are you still co... = No

Q43 Now that you have information from the patient history and physical examination, what **clinical syndrome, diagnosis, or health condition** are you considering as the **most probable** for George (you can write one only)?

____

Page Break

Display This Question:

If Now that you have the information from the patient history and physical examination, how importan... = Very important

Or Now that you have the information from the patient history and physical examination, how importan... = Important

Or Now that you have the information from the patient history and physical examination, how importan... = Uncertain

Or Now that you have information from the patient history and physical examination, are you still co... = No

Q44 Please indicate how confident you are that "**${Q43/ChoiceTextEntryValue}**" is the most probable clinical syndrome, diagnosis, or health condition for George:

- ◯ Not all confident (1)
- ◯ Not very confident (2)
- ◯ Somewhat confident (3)
- ◯ Confident (4)
- ◯ Very confident (5)

Page Break

Q57 **Part 3: Patient Treatment**

Please consider the **treatment approaches** that you would offer to George in the initial consultation and up to the end of your first follow up consultation. Please also consider your usual place of work when selecting the treatment approaches that you would offer.

58 Please select the **treatment approaches** that you would offer to George by the end of your first follow up consultation?

- ▢ Acupuncture (all types) (1)
- ▢ Advice and education (2)
- ▢ Cognitive behavioural therapy (3)
- ▢ Dry needling (4)
- ▢ Exercise and/or physical activity prescription (5)
- ▢ Electrotherapy (6)
- ▢ Manually applied technique/s that are aimed at soft tissue or joints (7)
- ▢ Orthopaedic footwear and/or or footwear recommendations (8)
- ▢ Orthotics (9)
- ▢ Provision of walking aids (14)
- ▢ Taping (10)
- ▢ Thermotherapy (cold/hot) (11)
- ▢ Weight management (12)
- ▢ Other (13) ____

Display This Question:

If Please select the treatment approaches that you would offer to George by the end of your first fo... = Advice and education

Q59 What **education-based strategies** would you use in the management of George by the end of your first follow up consultation? Please select up to 3.

- ▢ Activity pacing (9)
- ▢ Advice on over-the-counter medication (12)
- ▢ Advice on prescription medicine (13)
- ▢ Knowledge about condition (1)
- ▢ Knowledge about pain (2)
- ▢ Load management (controlling types of load, activity avoidance, and regression/progression) (11)
- ▢ Physiotherapy treatment options (3)
- ▢ Non-physiotherapy treatment options (4)
- ▢ Nutrition/healthy eating (7)
- ▢ Postural advice (8)
- ▢ Physical activity (5)
- ▢ Refer to other Health Care Professional (14)
- ▢ Skill building: pain coping, problem solving, positive thinking, goal setting (10)
- ▢ Sleep management (16)
- ▢ Weight loss/management (6)
- ▢ Other (15) ____

Display This Question:

If Please select the treatment approaches that you would offer to George by the end of your first fo... = Exercise and/or physical activity prescription

Q60 What form of **exercise therapy** would you use in the management of George by the end of your first follow up consultation? Please select up to 3:

- ☐ Aerobic Exercise For the purpose of this survey aerobic exercise refers to any moderate or vigorous activity defined as per the Exercise and Sports Science Australia, position statement, examples include running, swimming and cycling. Rate of perceived exertion >3-4/10. >60% maximum heart rate. (2)
- ☐ Aquatic exercise (10)
- ☐ Balance (6)
- ☐ General physical activity For the purpose of this survey general physical activity refers to any light physical activity define as per the Exercise and Sports and Science

Australia position statement, examples include gardening, walking, and cleaning. Rate of perceived exertion 1-2/10. 40<55% maximum heart rate. (11)

- ☐ Joint mobility (13)
- ☐ Neuromuscular exercise (3)
- ☐ Pilates (9)
- ☐ Plyometrics (4)
- ☐ Refer to health care professional (12)
- ☐ Strength Training (1)
- ☐ Stretching (5)
- ☐ Tai Chi (7)
- ☐ Yoga (8)
- ☐ Other (14) ____

Display This Question:

If Please select the treatment approaches that you would offer to George by the end of your first fo... = Weight management

Q61 How would you address **weight loss** as part of your management for George by the end of your first follow up consultation? Please select up to 3

- ▢ Exercise or physical activity advice for weight loss and/or maintenance (2)
- ▢ Nutritional advice for weight loss and/or maintenance (1)
- ▢ Refer to other health care professional (3)
- ▢ Refer to weight loss program/product (4)
- ▢ Other (5) ____

Display This Question:

If Please select the treatment approaches that you would offer to George by the end of your first fo... = Manually applied technique/s that are aimed at soft tissue or joints

Q62 What type of **manual therapy and/or what anatomical regions** would you treat as part of your management for George during the initial and follow up consultation? Please select up to 3

- ▢ Joint manual therapy thoracic spine (1)
- ▢ Joint manual therapy lumbar spine (2)
- ▢ Joint manual therapy sacroiliac joint (3)
- ▢ Joint manual therapy hip joint (4)
- ▢ Joint manual therapy knee/patellofemoral joint (5)
- ▢ Joint manual therapy ankle/foot joint (6)
- ▢ Soft tissue therapy thoracic spine region (7)
- ▢ Soft tissue therapy lumbar spine region (8)
- ▢ Soft tissue therapy gluteal muscles (9)
- ▢ Soft tissue therapy hip external rotator muscles (10)
- ▢ Soft tissue therapy hip adductor muscles (11)
- ▢ Soft tissue therapy quadriceps (12)
- ▢ Soft tissue therapy hamstring (13)
- ▢ Soft tissue therapy anterior/lateral compartment lower leg (14)
- ▢ Soft tissue therapy calve muscles (15)
- ▢ Other (16) ____

End of Block: Management

Start of Block: Attitudes Management

Start of Block: Block 10

Q66 Thank you for your participation in this survey. We greatly appreciate your time and effort. Your participation will help inform future research that aims to improve the management of people with chronic hip pain. The survey information sheet can again be viewed by clicking here Plain Language Summary

Q67 Would you like to go into draw to a win an Apple iPad Air? To go into the draw you will need to provide us with your contact name and email address.

- ◯ Yes (1)
- ◯ No (2)

Q68 Would you like to be provided with a summary of the results of this survey? Please note this may take up to 18 months. To receive a summary of the results you will need to provide us with your contact name and email address.

- ◯ Yes (1)
- ◯ No (2)

Q69 If you are happy to oblige, we can hold on to your name and contact details and contact you in the future if we have any research projects that we think you may be suitable for. We will not provide these details to anybody else.

◯ Yes (1)

◯ No (2)

Display This Question:

If Would you like to go into draw to a win an Apple iPad Air? To go into the draw you will need to p... = Yes

Or Would you like to be provided with a summary of the results of this survey? Please note this may... = Yes

Or If you are happy to oblige, we can hold on to your name and contact details and contact you in th... = Yes

Q70 Please state your first and last name:

____

Display This Question:

If Would you like to go into draw to a win an Apple iPad Air? To go into the draw you will need to p... = Yes

Or Would you like to be provided with a summary of the results of this survey? Please note this may... = Yes

Or If you are happy to oblige, we can hold on to your name and contact details and contact you in th... = Yes

Q71 Please provide your email address:

____

End of Block: Block 10
