# Supplementary material for: Clinical reasoning in managing chronic hip pain: One in two Australian and New Zealand physiotherapists diagnosed a case vignette with clinical criteria for hip OA as hip OA. A cross‐sectional survey
Source: Musculoskeletal Care. 2023 Mar 2;21(3):763–75. doi: 10.1002/msc.1751 (PMC10947065; doi:10.1002/msc.1751)
Supplement: Supplementary file 2 — Supplementary Material [file MSC-21-763-s004.pdf]

Part 1: Patient History

Primary presenting problem:
George, a 57-year-old man, presents to you at your current site of physiotherapy clinical practice. George reports that his main problem is pain at the outside of the left hip that extends into the groin and buttock and that this pain began 3 months ago (see pain diagram). Today George rates the intensity of his hip and groin pain as a 4 out of 10 (higher scores indicate worse pain).

George's hip and groin pain started around the time he began trying to get fit for an upcoming skiing holiday (in two months' time) by walking more. His route included some stair climbing to work his thighs. He first noticed this pain on the outside of his hip when going down steps and then going up as well, and eventually when walking on the flat. The pain then spread to include the whole hip area and groin.

George is finding his sleep quality is reduced. George wakes up through the night due to his hip and groin pain. Now he is feeling more tired and irritable. After getting out of bed in the morning George reports that his hip feels stiff for approximately 30 minutes.

George has not seen any other health professional with regard to this issue. George is unsure what is causing his pain or how it happened, but believes he must have damaged something as the pain hasn't gotten any better. He is concerned that his pain won't be better by his holiday.

Secondary presenting problem:
George has a five-year history of intermittent low back pain for which he has not previously sought care. Episodes usually last for one to two months and then resolve on their own. George's back pain returned about the same time that the pain spread into his whole hip area; his back pain has gradually worsened over the last three months rather than resolving as normal. George is worried about this as the combination of back and hip pain is really wearing him down. George rates his low back pain today as 3 out of 10.

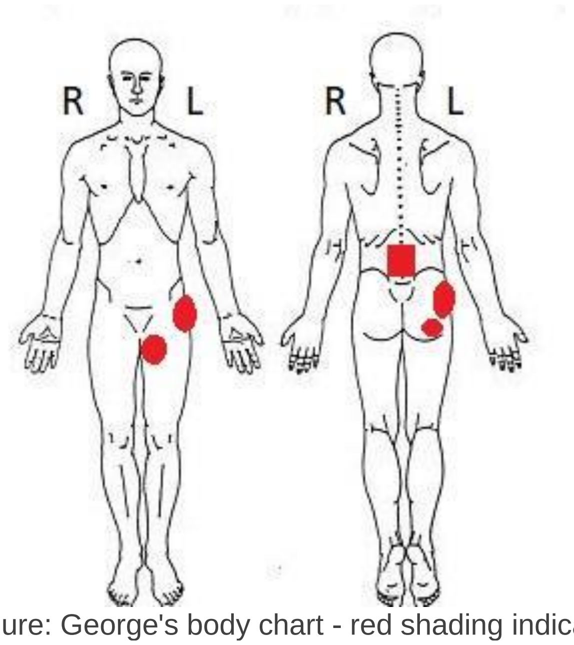

Figure: George's body chart - red shading indicates areas of pain distributions

Aggravating factors:

- George’s hip and groin pain increase immediately when ascending or descending stairs on the left leg. George has therefore been avoiding stairs.
- George cannot walk more than 15 minutes continuously. Pain gets progressively worse after 5 minutes of walking and can increase to a 6 out of 10. This causes George to stop walking (to sit down). Pain starts at the outside of the left hip then spreads into the groin and bottom. George’s back pain increases to a 4 out of 10 when walking. The low back pain does not change location.
- George’s hip and groin pain become more painful immediately when he puts his left shoe on. He has been avoiding this activity by wearing sandals or having his wife assist where necessary.

Easing factors:

- Sitting: once seated for 5 to 10 minutes after walking George’s hip and groin pain starts to lessen; it may take up to 1 hour for George's hip and groin pain to return to resting levels.
- George has tried taking Panadol tablets when the pain is worse but has found this ineffective at relieving his pain.

Medical history:
George reports that:

- he has not had any episodes of pain referral into the legs or any changes in his lower limb sensation or strength
- he has not experienced any changes with his urination and he has had no sensory disturbances around his perineal (saddle) or genital area
- he has no history of cancer
- he has not had any recent illness or fever
- he does not smoke or drink alcohol regularly
- he has not suffered from any mental illness
- he does not take any regular medications and has not ever had to take any regular medications
- he has not had any previous lower limb injuries

George believes he is slightly overweight. He reports that he is 170cm tall and weighs 85kg.

Physical activity and exercise:
George was walking 4km every day before the hip and groin pain started. Now he is only walking incidentally around the house and at work. George enjoyed exercising at a gym once a week where he lifted weights. He ceased going to the gym after the groin and hip pain started as he thought that lifting weights may have been making the pain worse.

Social history:
George is currently working full time in an office. He has not missed any work days and he is still working normal duties (mostly desk work). He is not concerned about his work at the moment. George lives with his wife and three children in their teens. George reports that his family is supportive, but he is worried he will let them down if he can’t go on their ski holiday.

Goals and expectations:
1. George is hoping you can help reduce his pain.
2. George would like to be able to ski with his family on their upcoming holiday.

Regarding treatment, George is unsure what to expect but is happy to try what you recommend.

Part 2: The physical examination

Presented below are the results of your physical examination with George. The series of questions that follow relate to this physical examination. Please consider your usual work-place setting.

Functional assessment

You complete a functional assessment in the following order. You allow George to sit in between each functional assessment.

Standing posture.

In weight-bearing position no resting fixed flexion deformity observed. No resting hip rotation deformity observed.

Taking shoes on and off:

You ask George to take on and off his (laced) shoes whilst seated on a chair of standard height. When attempting to lift his foot toward himself - moving into hip flexion and external rotation - George reports stiffness and a sudden increase of pain spreading through the left hip and into the groin and bottom. He rates this pain a 6 out of 10. To complete the movement, George lowers his left foot to the ground and bends toward his foot through his spine. George's back feels stiff whilst bending to get his shoe on; he rates his low back pain a 4 out of 10 during this movement.

Gait assessment:

You observe George walking indoors on a flat surface. George reports pain during the stance phase of the left leg: at the outside of the left hip, extending through the hip and into the groin and bottom. There is an obvious limp. George reports that his hip pain increases to a 4 out of 10 when he starts walking and his back feels stiff. After walking 100 metres his hip pain is a 5 out of 10 and his back pain is a 4 out of 10. You measure George's gait speed as 1 metre per second, walking at a comfortable self-selected pace, over a 20 metre segment at the start of the gait assessment. George reports that he could have kept walking but felt his hip and groin pain were likely to get worse. This is consistent with what he normally experiences when walking.

Stairs:

You ask George to ascend and descend 5 standard stairs (as he normally would). George initially ascends 2 steps by leading with the right leg and stepping to with the left leg. He also uses the handrail. You ask George to alternate leading with his left and right leg without using the handrail. George can position the left leg to step up and down, but upon weight-bearing he experiences worsening pain. The pain is at the outside of the left hip, extending through the hip, and into the groin and bottom. George’s pain increases as the assessment continues. After ascending then descending the 5 stairs George rates his pain a 6 out of 10. George reports that his back felt painful when stepping with either leg; the intensity of his low back pain increased to a 5 out of 10 by the end of the assessment.

Pain after functional assessments:

George has been seated and resting for approximately 3 minutes since completing the functional assessments. Compared to before the functional assessments George

reports that his hip and groin are now mildly more painful. The pain location hasn't changed but the intensity has increased to a 5 out of 10. George reports that his low back pain has increased to an intensity of 4 out of 10 and now feels stiffer whilst seated.

Lumbar assessment

Lumbar Flexion: standing

- 50 degrees active range of motion. George's fingertips - with straight arms - reached to his tibial tuberosity, 43cm from the ground. Stiffness and pain are the limiting factors. Pain is reported when moving through lumbar flexion at the left and right lumbar paraspinal region. No hip or groin pain reproduced.

Lumbar extension: standing

- 20 degrees active range of motion. Stiffness is the limiting factor. Pain is reported on the end of range at the limit of movement. No hip or groin pain reproduced.

Lateral flexion

- Left: 20 degrees active range of motion. Stiffness is the limiting factor. Pain is reported at the left paraspinal region and tightness is reported at the right paraspinal region; pain and stiffness reported on the end of range at the limit of movement.
- Right: 20 degrees active range of motion. Stiffness is the limiting factor. Pain is reported at the right paraspinal region and tightness is reported at the left paraspinal region; pain and stiffness reported on the end range at the limit of movement.

Lumbar quadrant test: extension, same side lateral flexion and rotation

- Left: pain on end of range at the limit of movement in the left paraspinal region. No hip or groin pain reproduced.
- Right: pain on end of range at the limit of movement in the right paraspinal region. No hip or groin pain reproduced.

Straight leg raise test:

- Left leg: 65 degrees active range of motion. George reports stiffness as the limiting factor in the hamstring and top of the calf. No hip or groin pain reproduced.
- Right leg: 70 degrees active range of motion. George reports stiffness as the limiting factor in the hamstring and top of the calf. No hip or groin pain reproduced.

Hip range of motion assessment:

Hip internal rotation range of motion: supine, in 90 degrees hip and knee flexion

- Left hip: 10 degrees active range of motion; passive range of motion no improvement. Both pain and stiffness are the limiting factors. Pain is reported at the left groin and buttock on the end of range at the limit of movement.
- Right hip: active and passive range of motion within normal ranges. No pain on movement.

Hip external rotation range of motion: supine in 90 degrees hip and knee flexion

- Left hip: 35 degrees active range of motion; passive range of motion no improvement. Both pain and stiffness are the limiting factors. Pain is reported at the left groin and buttock on the end of range at the limit of movement.

- Right hip active and passive range of motion within normal ranges. No pain on movement.

Hip abduction: supine

- Left hip: active and passive range of motion within normal ranges. Pain is reported at the left groin and buttock on the end of range at the limit of movement.

- Right hip: active and passive range of motion within normal ranges. No pain on movement.

Hip flexion: supine

- Left hip: 110 degrees active range of motion; passive range of motion no improvement. Both pain and stiffness are the limiting factors. Pain is reported at the left groin and buttock on the end of range at the limit of movement.

- Right hip: active and passive range of motion within normal ranges. No pain on movement.

Hip extension: prone

- Left hip: active and passive range of motion within normal ranges. No pain on movement.

- Right hip: active and passive range of motion within normal ranges. No pain on movement.

Tenderness on palpation:

- Painful to palpate left greater trochanter. Right side non-tender.
