# Supplementary material for: Clinical reasoning in managing chronic hip pain: One in two Australian and New Zealand physiotherapists diagnosed a case vignette with clinical criteria for hip OA as hip OA. A cross‐sectional survey
Source: Musculoskeletal Care. 2023 Mar 2;21(3):763–75. doi: 10.1002/msc.1751 (PMC10947065; doi:10.1002/msc.1751)
Supplement: Supplementary file 3 — Supplementary Material [file MSC-21-763-s005.pdf]

Supplementary digital content 3. Physiotherapists' ratings of importance for aspects of management of a hypothetical patient with chronic hip pain[1]

Question	Total respondents	Very important	Important	Uncertain	Unimportant	Very unimportant
**Survey Part A – after receiving patient history**						
Q18. At this point of the consultation, how important is it for you as the physiotherapist to determine which bodily structure(s) may be contributing to George's health problem?	220	100 (45%)	103 (47%)	5 (2%)	11 (5%)	1 (0%)
Q21. At this point of the consultation, how important is it for you as the physiotherapist to classify George's health problem as a specific clinical syndrome, diagnosis, or health condition?	220	21 (10%)	98 (44%)	22 (10%)	77 (35%)	2 (1%)
**Survey Part B**						
Q39. Now that you have the information from the patient history and physical examination, how important is it for you as the physiotherapist to determine which **bodily structure(s)** may contribute to George's health problem?	12 (people not previously identifying a bodily structure)	0 (0%)	3 (25%)	0 (0%)	9 (75%)	0 (0%)
Q42. Now that you have the information from the patient history and physical examination, **how important** is it for you as the physiotherapist to **categorise** George's health problem as a specific **clinical syndrome, diagnosis, or health condition**?	79 (respondents not classifying George's health problem after receiving Patient History)	3 (4%)	26 (33%)	8 (10%)	41 (52%)	1 (1%)

Q47. **How important** is it to you as the physiotherapist to **tell** the patient the **clinical syndrome, diagnosis, or health condition** that you have identified?	177 (respondents that diagnosed George's health problem)	53 (30%)	102 (58%)	15 (8%)	7 (4%)	0 (0%)

[1]Values are the number (percentage) unless otherwise stated. .
