# Supplementary material for: Clinical reasoning in managing chronic hip pain: One in two Australian and New Zealand physiotherapists diagnosed a case vignette with clinical criteria for hip OA as hip OA. A cross‐sectional survey
Source: Musculoskeletal Care. 2023 Mar 2;21(3):763–75. doi: 10.1002/msc.1751 (PMC10947065; doi:10.1002/msc.1751)
Supplement: Supplementary file 4 — Supplementary Material [file MSC-21-763-s003.pdf]

Supplementary digital content 4. Physiotherapists' ratings of confidence for aspects of management of a hypothetical patient with chronic hip pain. [1]

Question	Total participants responding	Very confident	Confident	Somewhat confident	Not very confident	Not at all confident
**Survey Part A**						
Q20. Please indicate how confident you are at this stage of the patient assessment, that the ____ is/are the most probable bodily structure(s) contributing to George's health problem?	208	36	102	64	6	0
Q23. Please indicate hoe confident you are at this stage of the patient assessment that the ____ is the most probable clinical syndrome, diagnosis, or health condition for George?	141	12 (9%)	59(42%)	63 (45%)	7 (5)	0 (0%)
**Survey Part B**						
Q33. Please indicate **how confident** you are that the_____ is/are the **most probable bodily structure(s)** contributing to George's health problem:	14	3 (21%)	5 (36%)	5 (36%)	1 (7%)	0 (%)
Q36. Please indicate how confident you are about your classification of ____ for George:	8	0 (0%)	4 (50%)	1 (1%)	3 (38%)	0 (0%)
Q41. Please indicate how confident you are that the ___ is/are the most probable bodily structure(s) contributing to George's health problem:	34	3 (9%)	18 (53%)	12 (35%)	1 (1%)	0 (0%)
Q44. Please indicate **how confident** you are that ____ is the most probable clinical syndrome, diagnosis, or health condition for George:	57	3 (5%)	27 (47%)	24 (42%)	3 (5%)	0 (0%)

[1]Values are the number (percentage) unless otherwise stated.
