# Supplementary material for: Clinical reasoning in managing chronic hip pain: One in two Australian and New Zealand physiotherapists diagnosed a case vignette with clinical criteria for hip OA as hip OA. A cross‐sectional survey
Source: Musculoskeletal Care. 2023 Mar 2;21(3):763–75. doi: 10.1002/msc.1751 (PMC10947065; doi:10.1002/msc.1751)
Supplement: Supplementary file 5 — Supplementary Material [file MSC-21-763-s002.pdf]

Supplementary digital content 5. Types of advice, exercise, manual therapy, and weight loss treatments offered[1]

Question	Respondents	Treatments	Respondents offering treatments
Q59. What education-based strategies would you use in the management of George by the end of your first follow up consultation? Please select up to 3.	217 (those selecting education as a treatment)	Activity pacing	57 (26%)
		Advice on the over-the-counter medication	24 (11%)
		Advice on prescription medicine	0 (0%)
		Knowledge about condition	168 (78%)
		Knowledge about pain	5 (2%)
		Load management (controlling types of load, activity avoidance, and regression/progression)	154 (71%)
		Physiotherapy treatment options	70 (32%)
		Non-physiotherapy treatment options	3 (1%)
		Nutrition/healthy eating	0 (0%)
		Postural advice	13 (6%)
		Physical activity	42 (19%)
		Refer to other Health Care Professional	3 (1%)
		Skill building: pain coping, problem solving, positive thinking, goal setting	12 (6%)
		Sleep management	18 (8%)
		Weight loss/management	12 (6%)
		**Other (please specify)**[1,2]	2 (1%)
		Guided by George’s expectations	1 (0%)

		Hydrotherapy	1 (0%)
Q.60 What form of exercise therapy would use in the in the management of George by the end of your first follow up consultation? Please select up to 3:	216 (those selecting advice and education as a treatment)	Aerobic exercise (For the purpose of this survey aerobic exercise refers to any moderate or vigorous activity defined as per the Exercise and Sports Science Australia, position statement, examples include running, swimming and cycling. Rate of perceived exertion >3-4/10. >60% maximum heart rate.	36 (17%)
		Aquatic exercise	62 (29%)
		Balance	12 (6%)
		General physical activity (For the purpose of this survey general physical activity refers to any light physical activity define as per the Exercise and Sports and Science Australia position statement, examples include gardening, walking, and cleaning. Rate of perceived exertion 1-2/10. 40<55% maximum heart rate).	92 (43%)
		Joint mobility	81 (38%)
		Neuromuscular exercise	69 (32%)
		Pilates	16 (7%)
		Plyometrics	0 (0%)
		Refer to heath care professional	3 (1%)
		Strength Training	150

		Stretching	41
		Tai Chai	0
		Yoga	2
		Other[1,2]	24 (11%)
		Core muscle program	2 (1%)
		Functional exercises (e.g. walking, stairs)	2 (1%)
		GLAD[3] program	2 (1%)
		Gluteal control exercises	2 (1%)
		Hydrotherapy classes	2 (1%)
		Isometric exercises	5 (2%)
		Patient-centred program (expectations, preferences, access, resources)	1 (<1%)
		Repeated movements	1 (<1%)
		Stationary bike	1 (<1%)
Q61. How would you address **weight loss** as part of your management for George by the end of your first follow up consultation? Please select up to 3	68 (those selecting weight loss as a treatment)	Exercise or physical activity advice for weight loss and/or maintenance	58 (85%)
		Nutritional advice for weight loss and/or maintenance	37 (54%)
		Refer to other health care professional	50 (74%)
		Refer to weight loss program/product	7 (10%)
		Other[1,]	6 (9%)
		Multidisciplinary care with dietician	3 (4%)

		Referral to General Practitioner for medication review	1 (1%)
		Advice about weight loss including specific strategies for weight loss	1 (1%)
62. What type of **manual therapy and/or what anatomical regions** would you treat as part of your management for George during the initial and follow up consultation? Please select up to 3:	168 (those selecting manual therapy as a treatment)	Joint manual therapy thoracic spine	2 (1%)
		Joint manual therapy lumbar spine	83 (49%)
		Joint manual therapy sacroiliac joint	10 (15%)
		Joint manual therapy hip joint	93 (55%)
		Joint manual therapy knee and patellofemoral joint	0 (0%)
		Joint manual therapy ankle/foot joint	0 (0%)
		Soft tissue thoracic spine region therapy	3 (2%)
		Soft tissue therapy lumbar spine region	51 (30.3%)
		Soft tissue therapy gluteal muscles	122 (73%)
		Soft tissue therapy deep hip external rotations	61 (36%)
		Soft tissue therapy hip adductors	23 (14%)
		Soft tissue therapy quadriceps	2 (2%)
		Soft tissue therapy hamstrings	3 (2%)
		Soft tissue therapy anterior/lateral compartment lower leg	2 (2%)
		Soft tissue therapy calve muscles	0 (0%)

	**Other (please specify):**	7 (4%)
	Contract-relax technique gluteal and iliopsoas muscle	1 (1%)

[1]Values are the number (percentage) unless otherwise stated. .
